# Recombinant human luteinizing hormone increases endometrial thickness in women undergoing assisted fertility treatments: a systematic review and meta-analysis

**DOI:** 10.3389/fphar.2024.1434625

**Published:** 2024-07-29

**Authors:** Routong Mao, Xiaohong Hou, Xiao Feng, Ruina Wang, Xiaofan Fei, Junzhao Zhao, Hui Chen, Jing Cheng

**Affiliations:** ^1^ Reproductive Center, Department of Obstetrics and Gynecology, The Second Affiliated Hospital and Yuying Children’s Hospital of Wenzhou Medical University, Wenzhou, China; ^2^ School of Life Sciences, Faculty of Science, University of Technology Sydney, Sydney, NSW, Australia

**Keywords:** IVF/ICSI-ET, r-hLH, r-hFSH, pregnancy rate, live birth rate

## Abstract

**Introduction:**

The optimal dosage of recombinant human luteinizing hormone (r-hLH) and its impact on endometrial thickness (EMT) when administered alongside recombinant human follicle-stimulating hormone (r-hFSH) during controlled ovarian stimulation (COS) for *in vitro* fertilization/intracytoplasmic sperm injection and embryo transfer are uncertain, which formed the aims of this systematic review and meta-analysis.

**Method:**

A search was performed in PubMed, Cochrane Library, Web of Science, EMBASE, CNKI, and Wanfang from its inception to 10 July 2023. Twenty-seven Randomized controlled trials comparing r-hFSH/r-hLH co-treatment with r-hFSH alone during *in vitro* fertilization/intracytoplasmic sperm injection and embryo transfer (IVF/ICSI-ET) were included. Pooled odds ratios (OR) for dichotomous data and mean differences (MD) for continuous data, with their respective 95% confidence intervals (CI), were generated. Meta-analysis employed fixed-effect or random-effect models based on heterogeneity, using Q-test and I2-index calculations. The main outcomes included EMT on trigger day, clinical pregnancy rate (CPR) and live birth rate (LBR).

**Results:**

r-hFSH/r-hLH significantly increased EMT on trigger day (MD = 0.27; 95% CI, 0.11–0.42; I^2^ = 13%), but reduced oocyte number (MD = −0.60; 95% CI, −1.07 to −0.14; I^2^ = 72%) and high-quality embryos (MD = −0.76; 95% CI, −1.41 to −0.10; I^2^ = 94%) than r-hFSH alone, more pronounced with the gonadotrophin-releasing hormone agonist long protocol. A subgroup analysis showed r-hLH at 75 IU/day increased CPR (OR = 1.23; 95% CI, 1.02–1.49; I^2^ = 16%) and EMT on trigger day (MD = 0.40; 95% CI, 0.19–0.61; I^2^ = 0%). Participants ≥35 years of age exhibited decreased retrieved oocytes (MD = −1.26; 95% CI, −1.78 to −0.74; I^2^ = 29%), but an increase in EMT on trigger day (MD = 0.26; 95% CI, 0.11–0.42; I^2^ = 29%).

**Conclusion:**

r-hFSH/r-hLH during COS significantly improved EMT compared to r-hFSH alone. An r-hLH dose of 75 IU/day may be considered for optimal pregnancy outcomes, which still require further clinical studies to support this dosing regime.

**Systematic Review Registration:**

[www.crd.york.ac.uk/PROSPERO], identifier [CRD42023454584].

## 1 Introduction

The “two-cell, two-gonadotropin” theory posits that luteinizing hormone (LH) and follicle-stimulating hormone (FSH) work synergistically to promote follicular growth and ovulation. During controlled ovarian stimulation (COS) for assisted reproductive technology (ART), recombinant human FSH (r-hFSH) stimulates the recruitment and growth of multiple follicles in the ovary. Simultaneously, gonadotropin-releasing hormone (GnRH) agonists or antagonists are administered daily to prevent premature LH surges or ovulation. FSH supplementation, without LH, can effectively induce follicle growth after the addition of GnRH agonists (GnRH-a) or antagonists (GnRH-A). This is because internal LH levels are adequate for supporting steroid production in ovarian cells. However, studies indicate that significantly low LH levels, falling below baseline, might detrimentally impact ART outcomes (Benmachiche et al., 2019).

A surge of LH and a successful ovulation lead to the formation of the corpus luteum. LH not only plays an important role in follicular growth, but also affects the decidualization of endometrium and embryo implantation. During COS, insufficient LH may lead to luteal phase deficiency (LPD) or luteal insufficiency, which may hinder proper endometrial preparation for implantation, potentially resulting in implantation failure, miscarriage, or uterine contractions, leading to embryo expulsion after transfer (Tesarik et al., 2020). However, A 3-year multicenter prospective randomized controlled trial (RCT) showed that adding recombinant human LH (r-hLH) during COS did not affect the live birth rate (LBR) or clinical pregnancy rate (CPR) in women with low endogenous LH levels (Lahoud et al., 2017); However, compared to r-hFSH supplementation alone, early r-hLH supplementation during ovarian stimulation notably increased CPR per cycle start and per embryo transfer in older women (Behre et al., 2015). The conflicting findings leave uncertainty about the necessity of exogenous LH in ART treatment.

To address this issue, several systematic review and meta-analyze papers have emerged. A paper compared r-hFSH and r-hLH regimens in COS against the human menopausal gonadotropin (hMG) regimen (Wang et al., 2020). Another one examined the effects of recombinant human chorionic gonadotropin (r-hCG) and r-hLH on final oocyte maturation in women with low fertility undergoing *in vitro* fertilization/intracytoplasmic sperm injection (IVF/ICSI) cycles (Youssef et al., 2016). These two and some others have all suggested that dual treatments can benefit pregnancy outcomes (Xiong et al., 2014; Youssef et al., 2016; Hua and Wang, 2023). However, the impact of such treatments on the endometrium remains inadequately investigated. Yet, the outcomes of IVF can be significantly influenced by endometrial factors.

Indeed, several studies have highlighted that endometrium thickness (EMT) is critical for a successful pregnancy during an ART cycle (Zhang et al., 2005; Esmailzadeh and Faramarzi, 2007; McWilliams and Frattarelli, 2007), whereas one study suggested the opposite (Shakerian et al., 2021). As mentioned above, although some studies have compared the efficacy of r-hFSH/r-hLH co-treatment with r-hFSH alone, most of them pay more attention to the effects on pregnancy outcome and follicular development, not the endometrial characteristics. In fact, few studies have performed in-depth comparisons of the effects of r-hFSH/r-hLH co-treatment on EMT and related pregnancy outcomes.

In addition, the protocol of additional r-hLH treatment (including the time and dose) may also affect the outcomes of the ART. While the effects of different timing for r-hLH addition have been studied (Hua and Wang, 2023), the impact of different r-hLH doses on clinical outcomes remains undetermined, which is also critical, as different daily doses of r-hLH can lead to distinct clinical outcomes. Excessive LH can negatively impact ART by inhibiting granulosa cells and follicular atresia, while insufficient LH leads to insufficient luteal function, which can adversely affect embryo implantation and pregnancy outcomes (Hill et al., 2012). Although LH supplementation during COS is often adjusted according to the patient’s follicular development and hormone levels, a comprehensive analysis of a large number of RCTs may suggest a dose regime that can significantly improve pregnancy outcomes in patients undergoing ART. Therefore, this study aimed to investigate the impact of r-hFSH/r-hLH co-treatment on EMT characteristics compared to r-hFSH alone. Furthermore, emphasis was placed on evaluating the optimal dose of r-hLH.

## 2 Materials and methods

### 2.1 Protocol and registration

We followed the Preferred Reporting Items for Systematic Reviews and Meta-Analysis (PRISMA) guidelines (PRISMA checklist presented in Supplementary Table S1). The research protocol was registered at http://www.crd.york.ac.uk/PROSPERO/ (registration number CRD42023454584). The inclusion criteria were: 1) women receiving IVF/ICSI; 2) the experimental group used r-hFSH and r-hLH during COS, with r-hFSH alone as control; 3) The outcomes included oocytes, embryo quality, EMT and IVF outcomes; 4) RCT. The exclusion criteria were: 1) not RCT, quasi-randomized trials, cohort or case-control studies, reviews, meta-analyses, case reports, animal or cell studies; 2) duplicate publication; 3) included women with severe gynecological diseases (e.g., intrauterine adhesions or endometriosis), any severe cardiovascular or cerebrovascular disease, or mental or neurological problems.

### 2.2 Search strategy

On 10 July 2023, six databases, PubMed, Cochrane Library, Web of Science, EMBASE, CNKI and Wan Fang, were searched using the search terms in combination, “Luteinizing Hormone”, “Fertilization *in Vitro*”, “Assisted Reproductive Techniques” and “Randomised Controlled Trial” (the details of the search strategy are shown in Supplementary Table S2).

### 2.3 Selection process and data extraction

RM and XF independently evaluated the abstracts and titles of every paper. In the event of a disagreement, a discussion was held with the third experimenter, RW, to reach a final consent.

A data extraction form was adapted from a published study (Shang et al., 2022). Any differences in extracted data were resolved between the experimenters through comparison and discussion. After verifying the accuracy, extracted data were entered into Review Manager 5.4.1 (Clarivate Analytics, PA, United States), including authors, year of publication, randomization method, age, COS protocol, number of patients, fertilization methods, r-hLH dosage, oocyte, embryo and IVF outcomes. The primary measurements for the meta-analysis were CPR, LBR and EMT on trigger day. The secondary measurements were implantation rate (IR), miscarriage rate (MR), high-quality embryo rate, number of oocytes retrieved, mature (MII) oocytes, and high-quality embryos. All measurements were defined according to the 2017 International Glossary of Infertility and Reproductive Care (Zegers-Hochschild et al., 2017). IR is defined as the number of gestational sacs observed divided by the number of embryos transferred. MR is defined as the number of patients who had a miscarriage divided by the number of patients who had a successful clinical pregnancy. CPR is defined as the number of clinical pregnancies expressed per 100 initiated cycles, aspiration cycles or embryo transfer cycles. LBR is defined as the number of deliveries that resulted in at least one live birth, expressed per 100 cycle attempts. The authors of the original studies were contacted when clarification was needed.

### 2.4 Assessment of risk of bias in included studies

Cochrane bias risk assessment tool (Sterne et al., 2019) was used for six domains: 1) random sequence generation (selection bias); 2) allocation concealment (selection bias); 3) blinding of participants and personnel (performance bias); 4) blinding of outcome assessment (detection bias); 5) incomplete outcome data (attrition bias) and 6) selective reporting (reporting bias). RM and XF independently evaluated each study. When discrepancies occurred, a discussion was held with the third experimenter, RW, to reach a final consent.

### 2.5 Statistical analysis

Review manager 5.4.1 was used for statistical analysis. Dichotomous data were expressed as an odds ratio (OR), and continuous data were expressed as mean difference (MD) with a 95% confidence interval (CI). Q-text and *I* ^2^-index were used to calculate heterogeneity and reported as a P-value and percentage, respectively. If there is no statistical heterogeneity, a fixed-effect model was used to calculate MD and OR with a 95% CI. If there is significant heterogeneity, a random-effect model was used. P values <0.05 were considered statistically significant.

### 2.6 Subgroup and sensitivity analysis

Subgroup analyses were performed for primary outcomes (CPR, LBR and EMT) and some secondary outcomes (IR, MR, the number of oocytes retrieved and MII oocytes retrieved) based on ovarian stimulation protocol, r-hLH dosage and the participant’s age. Sensitivity analysis was performed (Stata17.0, StataCorp LP, College Station, TX, United States) to examine the stability of the conclusions.

### 2.7 Publication bias assessment

We generated funnel plots for publication bias. If the asymmetry in the funnel plot is detected, it indicates that there is suspected publication bias in the research results, and the Egger’s test is further conducted to confirm whether publication bias exists. In addition, we evaluated the characteristics of the trial to detect whether this asymmetry may be due to publication bias or other factors, such as the methodological or clinical heterogeneity of the trial.

### 2.8 Assessing the certainty of evidence

The overall quality of evidence (QoE) was assessed independently by two experimenters (MR and FX) based on the risk of bias, inconsistency, indirection, inaccuracy, or publication bias according to the GRADE (Grading of Recommendations Assessment, Development and Evaluation) guidelines (Guyatt et al., 2008). Any disagreements between the two review authors were resolved by discussion, involving a third experimenter (RW) if needed. The QoE was classified as high, moderate, low, and very low.

## 3 Results

### 3.1 Study characteristics

**FIGURE 1:**
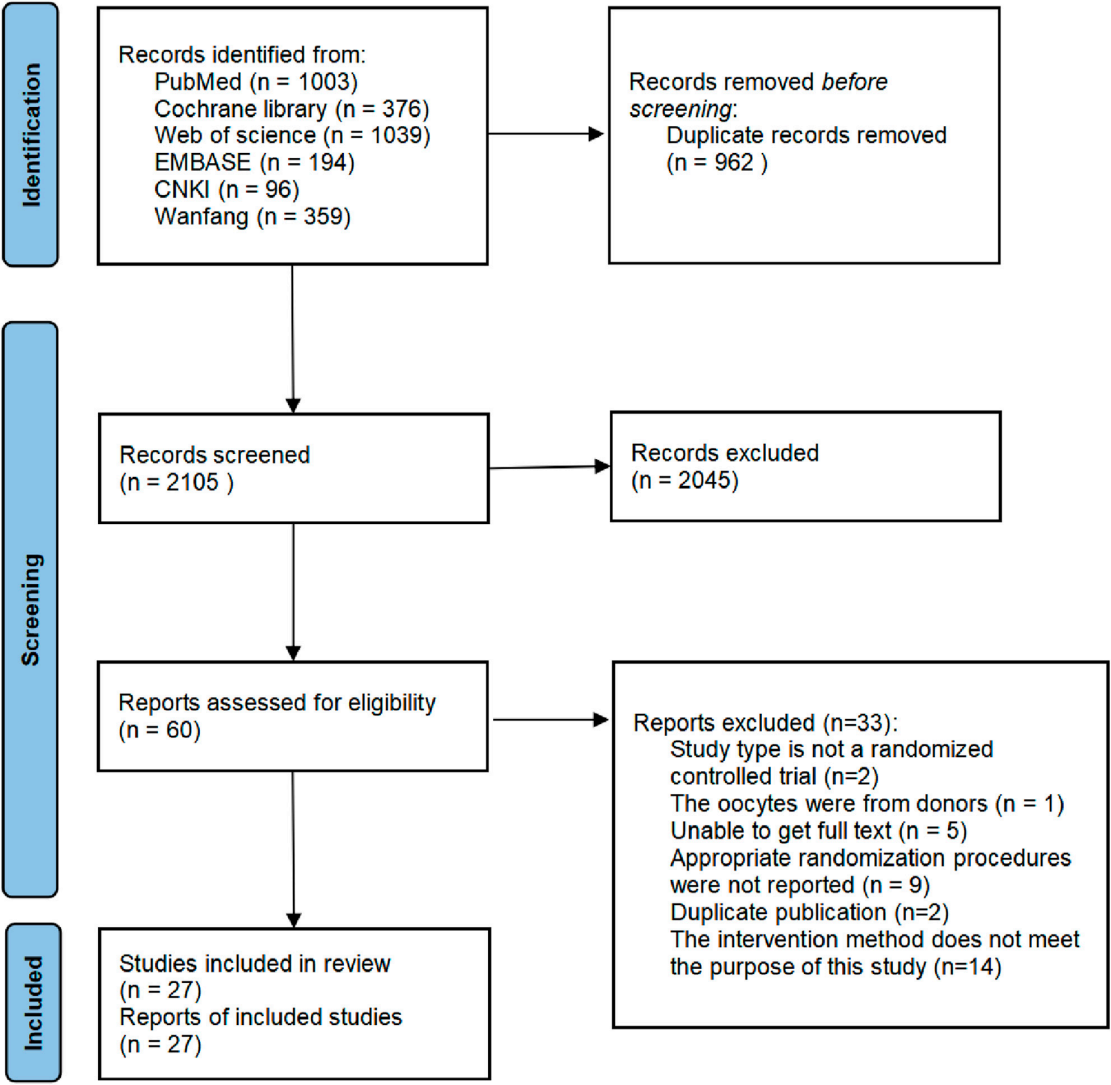
Flowchart of study design. Flowchart of study selection for systematic review and meta-analysis to compare the effects of recombinant human luteinizing hormone (r-hLH)/recombinant human follicle-stimulating hormone (r-hFSH) co-treatment during ovarian stimulation with r-hFSH treatment on women undergoing *in vitro* fertilization/intracytoplasmic sperm injection (IVF/ICSI).

A total of 3,067 papers were retrieved from six databases, including 962 duplicates. Then, 2045 papers were excluded due to unrelated topics; 33 papers were excluded for other reasons, as shown in Figure 1. A total of 27 studies were included and listed in Table 1. The detailed characteristics of these studies are shown in Table 1. Most studies adopted a single dose of r-hLH in their RCTs. There are two studies that employed multiple dose regimes. In the study of Fábregues et al. (2011), two doses of r-hLH were 37.5 IU/day and 75 IU/day in different groups. The values in Table 1 were listed in the order of group A, group B and group C. In the study by Hong et al. (2012), in addition to the control group (group C), patients in groups T1 (75IU/day) and T2 (150IU/day) were given r-hLH on the sixth day of r-hFSH ovulation induction, while patients in groups T3 (75IU/day) and T4 (150IU/day) were given r-hLH on the first day of r-hFSH ovulation induction. Thus, more than two data sets were presented for this study.

**TABLE 1:** Characteristics of trials included in the meta-analysis.

No.	Included RCTs	Method of randomization	Age (r-hFSH + r-hLH; r-hFSH)	Ovarian stimulation protocol	No. of patients (r-hFSH + r-hLH; r-hFSH)	Fertilization	r-hLH dosage	Outcomes
1	Berkkanoglu et al. (2007)	Computer-generated randomization sequence	36.3 ± 0.76; 34.9 ± 0.5	Microflare stimulation	46; 51	ICSI	75 IU/day	• No. of MII oocytes retrieved
								• CPR
2	Caserta et al. (2011)	Sealed envelopes	34.3 ± 3.5; 34.8 ± 3.6	GnRH-a long	498; 501	ICSI	75 IU/day	• No. of oocytes retrieved and MII oocytes retrieved
								• IR and CPR
3	Cédrin-Durnerin et al. (2004)	Sealed envelopes	31.4 ± 3.9; 31.7 ± 3.8	GnRH-A	107; 96	IVF or ICSI	75 IU/day	• No. of oocytes retrieved and MII oocytes retrieved
								• IR, CPR and MR
4	De Placido et al. (2005)	Computer-generated randomization sequence	31.5 ± 3.9; 30.4 ± 4.1	GnRH-a long	59; 58	IVF or ICSI	150 IU/day	• No. of MII oocytes retrieved’
								• IR, MR and CPR
5	Fábregues et al. (2006)	Computer-generated randomization sequence	38.4 ± 1.4; 38.2 ± 1.5	GnRH-a long	60; 60	IVF or ICSI	150 IU/day	• No. of oocytes retrieved, MII oocytes retrieved and high-quality embryos
								• EMT
								• IR, MR and CPR
6	Fábregues et al. (2011)	Computer-generated randomization sequence	37.3 ± 0.3; 37.7 ± 1.8; 37.6 ± 0.4	GnRH-a long	62; 63; 62	IVF or ICSI	37.5 IU/day; 75 IU/day	• No. of oocytes retrieved, MII oocytes retrieved and high-quality embryos
								• EMT
								• IR, CPR and MR
7	Griesinger et al. (2005)	Sealed envelopes	30.3 ± 4.7; 30.5 ± 4.2	GnRH-A	54; 54	IVF or ICSI	75 IU/day	• IR and CPR
8	Humaidan et al. (2004)	Computer-generated randomization sequence	30.8 ± 3.9; 30.5 ± 4.0	GnRH-a long	116; 115	IVF or ICSI	doses of r-hFSH and r-hLH were given in a ratio of 2:1	• No. of oocytes retrieved
								• IR and CPR
9	Humaidan et al. (2017)	Interactive voice response system	38.3 ± 2.9; 38.3 ± 3.0	GnRH-a long	462; 477	IVF or ICSI	150 IU/day	• No. of MII oocytes retrieved
								• IR, CPR and LBR
10	König et al. (2013)	Sealed envelopes	38.0 ± 1.9; 37.9 ± 2.0	GnRH-A	125; 128	IVF or ICSI	150 IU/day	• No. of oocytes retrieved
								• EMT
								• IR, MR and CPR
11	Lahoud et al. (2017)	Computer-generated randomization sequence	34.0 ± 4.51; 35.2 ± 3.78	GnRH-a long	43; 57	IVF or ICSI	75 IU/day	• No. of oocytes retrieved and high-quality embryos
								• MR, CPR and LBR
12	Balasch et al. (2001)	Computer-generated randomization sequence	34.8 ± 0.8; 33.6 ± 0.8	GnRH-a long	15; 13	IVF or ICSI	75 IU/day	• No. of oocytes retrieved and MII oocytes retrieved
13	Levi-Setti et al. (2006)	Computer-generated randomization sequence	32.2 ± 2.46; 32.3 ± 2.30	GnRH-A	20; 20	ICSI	75 IU/day	• No. of oocytes retrieved, MII oocytes retrieved and high-quality embryo rate
								• IR and CPR
14	Lisi et al. (2005)	Computer-generated randomization sequence	35.2 ± 0.4: 35.8 ± 0.3; 36.1 ± 0.6	GnRH-a long	109; 179; 240	IVF or ICSI	37.5 IU/day; 75 IU/day	• CPR
15	Lisi et al. (2012)	Computer-generated randomization sequence	33.6 ± 3.4; 32.8 ± 3.8	GnRH-a long	75; 75	IVF or ICSI	75 IU/day	• No. of oocytes retrieved, MII oocytes retrieved and high-quality embryo rate
								• IR and CPR
16	Marrs et al. (2004)	Computer-generated randomization sequence	32.4 ± 3.8; 31.9 ± 3.7	GnRH-a long	212; 219	ICSI	150 IU/day	• No. of oocytes retrieved, MII oocytes retrieved
								• IR and CPR
17	Matorras et al. (2009)	Computer-generated randomization sequence	36.6 ± 1.6; 36.7 ± 1.5	GnRH-a long	63; 68	ICSI	150 IU/day	• No. of oocytes retrieved, MII oocytes retrieved
								• IR, CPR and LBR
18	Musters et al. (2012)	Computer-generated randomization sequence	38.3 ± 5.7; 38.6 ± 2.4	GnRH-a long	116; 128	IVF or ICSI	adjusted according to AFC	• No. of oocytes retrieved and high-quality embryos
								• MR and CPR
19	NyboeAndersen et al. (2008)	Sealed envelope	31.72 ± 3.87; 31.8 ± 3.98	GnRH-a long	265; 261	IVF or ICSI	75 IU/day or 150 IU/day according to age	• No. of oocytes retrieved
								• IR
20	Pezzuto et al. (2010)	Computer-generated randomization sequence	35 ± 4.0; 34 ± 4.2	GnRH-a long	40; 40	ICSI	75 IU/day	• No. of oocytes retrieved
								• CPR
21	Razi et al. (2014)	Random numbers table	31.85 ± 1.59; 31.35 ± 1.69	GnRH-a long	20; 20	IVF or ICSI	75 IU/day	• No. of oocytes retrieved, MII oocytes retrieved
								• EMT
								• IR, MR and CPR
22	Sauer et al. (2004)	Computer-generated randomization sequence	32.26 ± 4.0	GnRH-A	21; 21	ICSI	150 IU/day	• IR and CPR
23	Li (2012)	Random numbers table	32.59 ± 4.44; 31.67 ± 3.72	GnRH-a long	22; 24	IVF or ICSI	75 IU/day	• No. of retrieved oocytes and high-quality embryo rate
								• EMT
								• IR, MR and CPR
24	Tarlatzis et al. (2006)	Computer-generated randomization sequence	30.5 ± 3.5; 30.3 ± 3.6	GnRH-a long	55; 59	IVF or ICSI	75 IU/day	• No. of oocytes retrieved, MII oocytes retrieved
								• MR, CPR and LBR
25	Vuong et al. (2015)	Computer-generated randomization sequence	38 (36, 40); 38 (36, 40)	GnRH-A	120; 120	IVF	75 IU/day	• No. of oocytes retrieved and high-quality embryos
								• EMT
								• MR, CPR and LBR
26	Hong et al. (2012)	Computer-generated randomization sequence	36.0 ± 1.2; 36.6 ± 1.5; 36.6 ± 1.4; 36.5 ± 14.2; 36.2 ± 1.3	GnRH-a long	63; 65; 68; 63; 61	IVF or ICSI	75 IU/day; 150 IU/day; 75 IU/day; 150 IU/day	• No. of oocytes retrieved and MII oocytes retrieved
								• IR, MR and CPR
27	Younis et al. (2016)	Sealed envelopes	38.9 ± 2.8; 38.6 ± 3.7	GnRH-A	32; 30	IVF or ICSI	doses of r-hFSH and r-hLH were given in a ratio of 2:1	• No. of oocytes retrieved, MII oocytes retrieval and high-quality embryo rate
								• EMT
								• CPR

RCTs, randomized controlled trials; r-hFSH, recombinant human follicle-stimulating hormone; r-hLH, recombinant human luteinizing hormone; GnRH-A, GnRH, antagonist; GnRH-a, GnRH, agonist; MII, metaphase II; EMT, endometrial thickness; IR, implantation rate; MR, miscarriage rate; CPR, clinical pregnancy rate; LBR, live birth rate; IVF, *in vitro* fertilization; ICSI, intracytoplasmic sperm injection.

### 3.2 Risk of bias

**FIGURE 2:**
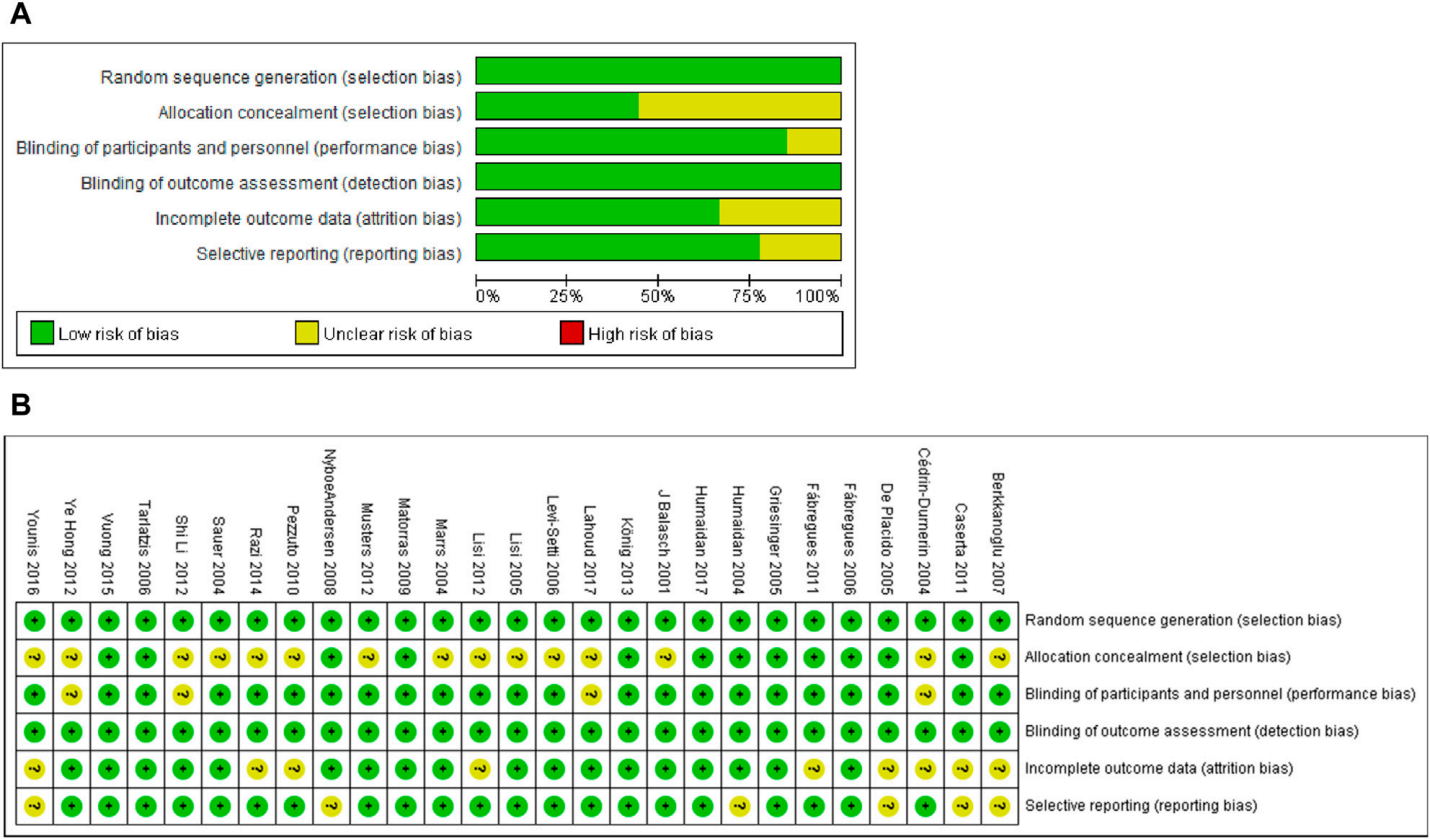
Risk of bias assessment of included studies. **(A)** Risk of bias graph. Assessors’ judgement of each risk of bias item was expressed as a percentage across all included studies. **(B)** Risk of bias summary. Assessors’ judgment on each bias risk item included in each study.

*Sequence generation*. All 27 studies used appropriate randomization methods (the specific methods used in each study were listed in Table 1) and were deemed low risk (Figure 2).

*Allocation concealment*. Fifteen studies used appropriate methods to conceal the allocation and were deemed low risk. Twelve studies did not adequately describe allocation concealment and were rated as unclear risks.

*Performance bias*. Four studies did not report blinding, and therefore, they were rated as an unclear risk. Twenty-three studies used double-blind or triple-blind and were deemed low risk.

*Detection bias*. Five studies lacked information on the blinding of outcome assessors or were inadequately described. Since the primary outcomes (CPR, LBR and EMT) of these studies were objective and were unlikely to cause detection bias. Thus, all 27 studies included were identified as low risks.

*Incomplete outcome data*. Nine studies were rated as having an unclear risk of bias because they did not provide sufficient information for us to make conclusive judgments. Eighteen studies were considered low risk because the proportion of loss of follow-up and the reasons for loss of follow-up in each treatment group were similar.

*Selective reporting*. Six studies were rated as having an unclear risk of bias because there was not enough information described in the Methods section. The remaining twenty-one studies were identified as low risk since the results of all the results for methods described in the method were reported.

### 3.3 Synthesis of results

**FIGURE 3:**
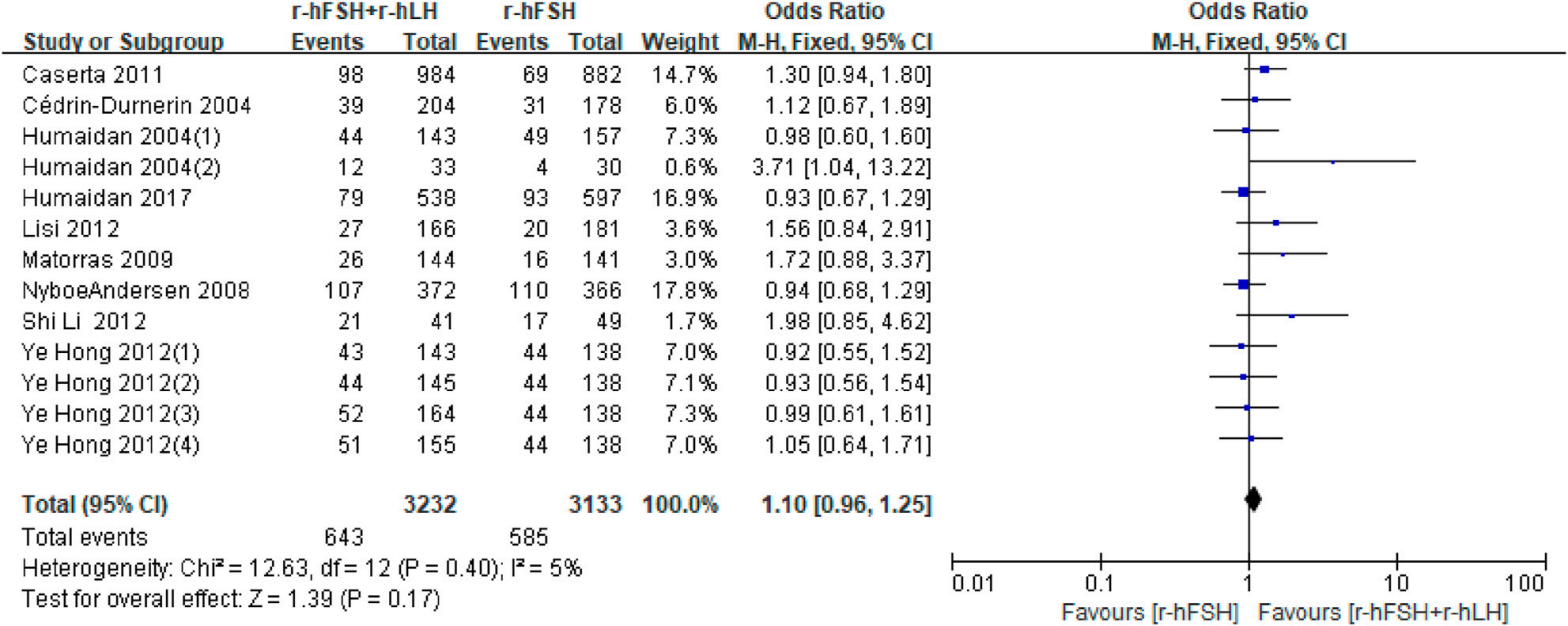
Forest plot of IR. r-hFSH/r-hLH co-treatment (r-hFSH + r-hLH) *versus* r-hFSH treatment (r-hFSH).

**IR.** IR was reported in eighteen studies (Table 1). Nine studies were unavailable for meta-analysis due to insufficient data (Marrs et al., 2004; De Placido et al., 2005; Griesinger et al., 2005; Fábregues et al., 2006; Levi-Setti et al., 2006; Fábregues et al., 2011; König et al., 2013; Razi et al., 2014). Overall, the results of the meta-analysis of the remaining nine studies showed that there was no significant difference in IR between r-hFSH/r-hLH co-treatment and r-hFSH alone during COS (OR = 1.10; 95% CI, 0.96–1.25; P= 0.17) (Figure 3). The QoE was moderate due to publication bias (Table 2). In the subgroup analysis, no differences were observed (Table 3).

**TABLE 2:** Clinical outcomes.

Outcomes	Anticipated absolute effects (95% CI)		Relative effect (95% CI)	No. of participants (studies)	Certainty of the evidence (GRADE)
	Assumed risk	Corresponding risk			
	r-hFSH	r-hFSH + r-hLH			
Implantation rate	187 per 1,000	199 per 1,000 (100–512)	OR 1.10 (0.96–1.25)	3,545 (9 RCTs)	⊕⊕⊕㊀ Moderate^a^
Clinical pregnancy rate	249 per 1,000	268 per 1,000 (107–789)	OR 1.09 (0.96–1.24)	5,631 (23 RCTs)	⊕⊕⊕⊕ High
Miscarriage rate	151 per 1,000	157 per 1,000 (94–333)	OR 1.12 (0.73–1.72)	2010 (10 RCTs)	⊕⊕⊕⊕ High
Live birth rate	140 per 1,000	131 per 1,000 (106–233)	OR 0.94 (0.70–1.27)	1,524 (5 RCTs)	⊕⊕⊕⊕ High
EMT on trigger day	-	MD 0.27 higher (0.11 higher to 0.42 higher)	-	708 (6 RCTs)	⊕⊕⊕⊕ High
No. of oocytes retrieved	-	MD 0.93 lower (1.07 lower to 0.14 lower)	-	3,510 (17 RCTs)	⊕⊕㊀㊀ Low^a^ ^,^ ^b^
No. of MII oocytes retrieved	-	MD 0.16 lower (0.63 lower to 0.31 higher)	-	2,975 (13 RCTs)	⊕⊕㊀㊀ Low^a^ ^,^ ^b^
No. of high-quality embryos	-	MD 0.76 lower (1.41 lower to 0.1 lower)	-	438 (3 RCTs)	⊕⊕㊀㊀ Low^b^ ^,^ ^c^

^a^ Downgraded one level due to publication bias.

^b^ Downgraded one level due to serious inconsistency with unexplained heterogeneity.

^c^ Downgraded one level due to serious imprecision: small studies and/or wide confidence interval. CI, confidence interval; MD, mean difference; OR, odds ratio; EMT, endometrium; MII, metaphase II; OR, odds ratio; r-hFSH, recombinant-human follicle-stimulating hormone; r-hLH, recombinant-human luteinizing hormone.

**TABLE 3:** Effect estimate and heterogeneity of subgroup analysis for IR.

Subgroup	No. of studies (n)	No. of women (n)	Effect estimate OR/MD (95% CI)	*I* ^*2*^	P
Ovarian stimulation protocol					
GnRH-a long protocol	8	3,342	1.09 (0.96–1.25)	13%	0.19
GnRH-A protocol	1	203	1.12 (0.67–1.89)	NA	0.67
r-hLH dosage					
75 IU/day	5	1,651	1.20 (0.99–1.47)	0%	0.07
150 IU/day	3	1,320	1.03 (0.82–1.28)	0%	0.83
75 IU/day or 150 IU/day according to age	1	526	0.94 (0.68–1.29)	NA	0.70
keep the ratio of r-hFSH:r-hLH to 2:1	3	231	1.19 (0.76–1.86)	73%	0.45
Average age of participants in both groups					
≤35	6	2,116	1.15 (0.96–1.37)	1%	0.12
>35	4	1,429	1.04 (0.86–1.25)	13%	0.70

CI, confidence interval; MD, mean difference; OR, odds ratio; GnRH, gonadotrophin releasing hormone; GnRH-A, GnRH, antagonist; GnRH-a, GnRH, agonist; r-hFSH, recombinant human follicle-stimulating hormone; r-hLH, recombinant human luteinizing hormone; NA, not available.

**FIGURE 4:**
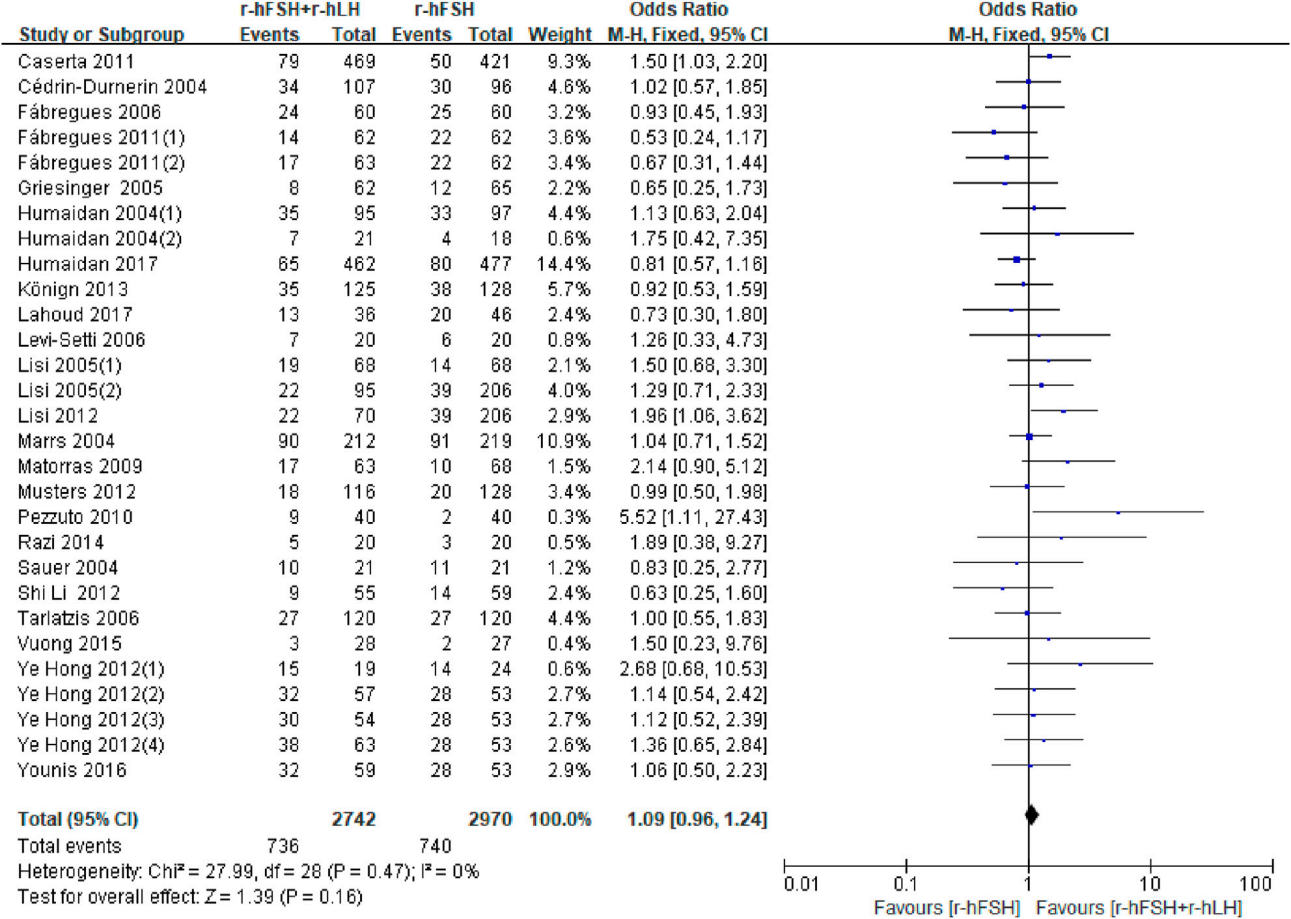
Forest plot of CPR. r-hFSH/r-hLH co-treatment (r-hFSH + r-hLH) *versus* r-hFSH treatment (r-hFSH).

**CPR.** Twenty-five studies reported CPR (Table 1). Except for the study by Berkkanoglu et al. (2007) and De Placido et al. (2005), in which the data were unavailable for analysis, no significant difference was found between the r-hFSH/r-hLH co-treatment and r-hFSH treatment (OR = 1.09; 95% CI, 0.96–1.24; P= 0.16) (Figure 4), with a high QoE (Table 2). Subgroup analysis showed that r-hLH supplementation at 75 IU/day significantly increased the CPR while other dosages of r-hLH did not (OR = 1.23; 95% CI, 1.02–1.49; p = 0.03) (Table 4).

**TABLE 4:** Effect estimate and heterogeneity of subgroup analysis.

Outcomes	Subgroup	No. of studies (n)	No. of women (n)	Effect estimate OR/MD (95% CI)	*I* ^*2*^	P
CPR						
	Ovarian stimulation protocol					
	GnRH-a long protocol	16	4,660	1.13 (0.98–1.29)	19%	0.09
	GnRH-A protocol	7	971	0.95 (0.71–1.28)	0%	0.75
	r-hLH dosage					
	75 IU/day*	15	2,917	1.23 (1.02–1.49)	16%	0.03
	150 IU/day	8	2,189	0.99 (0.80–1.19)	0%	0.82
	37.5 IU/day	1	474	0.53 (0.24–1.17)	NA	0.12
	keep the ratio of r-hFSH:r-hLH to 2:1	3	293	1.23 (0.73–2.07)	0%	0.44
	adjusted according to AFC	1	244	0.99 (0.50–1.98)	NA	0.98
	Average age of participants in both groups					
	≤35	12	2,468	1.20 (0.99–1.46)	3%	0.06
	>35	12	3,163	1.02 (0.87–1.20)	0%	0.81
EMT on trigger day						
	Ovarian stimulation protocol					
	GnRH-a long protocol*	4	393	0.26 (0.10–0.42)	37%	0.002
	GnRH-A protocol	2	315	0.33 (−0.14–0.80)	0%	0.17
	r-hLH dosage					
	75 IU/day**	3	211	0.40 (0.19–0.61)	0%	0.0002
	150 IU/day	2	373	0.36 (−0.04–0.77)	0%	0.08
	37.5 IU/day	1	125	0.00 (−0.28 to 0.28)	NA	1.00
	keep the ratio of r-hFSH:r-hLH to 2:1	1	62	−0.10 (−1.38 to 1.18)	NA	0.88
	Average age of participants in both groups					
	≤35	2	86	0.38 (−0.51–1.27)	19%	0.41
	>35**	4	622	0.26 (0.11–0.42)	29%	0.0009
No. of oocytes retrieved						
	Ovarian stimulation protocol					
	GnRH-a long protocol*	13	2,952	−0.76 (−1.26 to −0.26)	74%	0.003
	GnRH-A protocol	4	558	0.04 (−0.75–0.82)	0%	0.93
	r-hLH dosage					
	75 IU/day	11	1925	−0.34 (−0.98 to 0.30)	66%	0.30
	150 IU/day**	4	935	−1.15 (−1.82 to −0.49)	38%	0.0007
	37.5 IU/day*	1	125	−1.60 (−2.75 to −0.45)	NA	0.007
	75 IU/day or 150 IU/day according to age	1	526	−0.70 (−1.52 to 0.12)	NA	0.09
	keep the ratio of r-hFSH:r-hLH to 2:1	1	62	0.20 (−1.72–2.12)	NA	0.84
Average age of participants in both groups						
	≤35	11	2,657	−0.34 (−0.89 to 0.20)	60%	0.22
	>35**	6	853	−1.26 (−1.78 to −0.74)	29%	<0.00001

CI, confidence interval; MD, mean difference; OR, odds ratio; GnRH, gonadotrophin releasing hormone; GnRH-A, GnRH, antagonist; GnRH-a, GnRH, agonist; r-hFSH, recombinant human follicle-stimulating hormone; r-hLH, recombinant human luteinizing hormone; AFC, antral follicle; CPR, clinical pregnancy rate; EMT, endometrium thickness; NA, not available; *, P< 0.05; **, P< 0.001.

**FIGURE 5:**
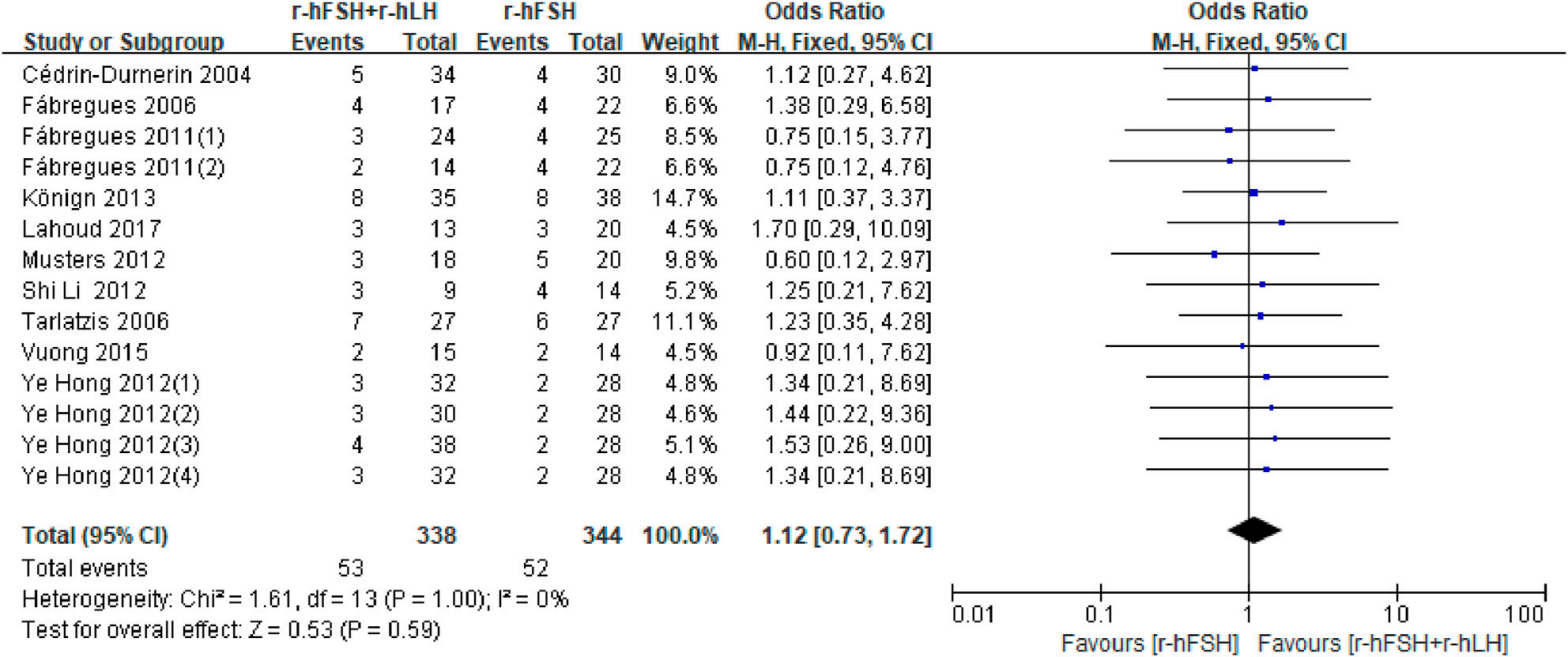
Forest plot of MR. r-hFSH/r-hLH co-treatment (r-hFSH + r-hLH) *versus* r-hFSH treatment (r-hFSH).

**MR.** Twelve studies assessed MR (Table 1). Two studies could not be included in the meta-analysis because there was no data available for the calculations (De Placido et al., 2005; Razi et al., 2014). Two studies (De Placido et al., 2005; Razi et al., 2014) showed no difference between the r-hFSH/r-hLH co-treatment and r-hFSH alone, therefore was not included in the meta-analysis. The meta-analysis of the remaining ten studies showed no difference in the MR between the combination of r-hFSH and r-hLH during ovulation induction compared with r-hFSH alone (OR = 1.12; 95% CI, 0.73–1.72; P= 0.59) (Figure 5). The QoE was high (Table 2). No difference was observed in the subgroup analysis (Table 5).

**TABLE 5:** Effect estimate and heterogeneity of subgroup analysis for MR.

Subgroup	No. of studies (n)	No. of women (n)	Effect estimate OR/MD (95% CI)	*I* ^*2*^	P
Ovarian stimulation protocol					
GnRH-a long protocol	7	1,131	1.11 (0.65–1.88)	0%	0.70
GnRH-A protocol	3	696	1.15 (0.56–2.35)	0%	0.70
r-hLH dosage					
75 IU/day	3	1,081	1.29 (0.72–2.29)	0%	0.39
150 IU/day	2	718	1.10 (0.52–2.32)	0%	0.81
37.5 IU/day	1	125	0.75 (0.12–4.76)	NA	0.76
adjusted according to AFC	1	244	0.60 (0.12–2.97)	NA	0.53
Average age of participants in both groups					
≤35	3	363	1.11 (0.41–2.97)	0%	0.84
>35	7	1,464	1.13 (0.70–1.80)	0%	0.62

CI, confidence interval; MD, mean difference; OR, odds ratio; GnRH, gonadotrophin releasing hormone; GnRH-A, GnRH, antagonist; GnRH-a, GnRH, agonist; AFC, antral follicle; NA, not available.

**FIGURE 6:**
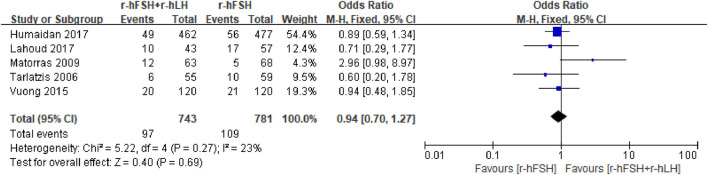
Forest plot of LBR. r-hFSH/r-hLH co-treatment (r-hFSH + r-hLH) *versus* r-hFSH treatment (r-hFSH).

**LBR.** Five studies, including 1,524 participants, reported the effect on LBR, which were used for meta-anlysis (Table 1). The results indicated that the addition of r-hLH did not improve LBR in women undergoing IVF/ICSI (OR = 0.94, 95% CI: 0.70–1.27; p = 0.69; *I* ^*2*^ = 23%) (Figure 6). According to GRADE system, the QoE was high (Table 2). No difference was observed in the subgroup analysis (Table 6).

**TABLE 6:** Effect estimate and heterogeneity of subgroup analysis for LBR.

Subgroup	No. of studies (n)	No. of women (n)	Effect estimate OR/MD (95% CI)	*I* ^*2*^	P
Ovarian stimulation protocol					
GnRH-a long protocol	4	1,284	0.94 (0.68–1.31)	42%	0.72
GnRH-A protocol	1	240	0.94 (0.48–1.85)	NA	0.86
r-hLH dosage					
75 IU/day	3	454	0.79 (0.49–1.29)	0%	0.35
150 IU/day	2	1,070	1.04 (0.72–1.52)	75%	0.82
Average age of participants in both groups					
≤35	1	114	0.60 (0.20–1.78)	NA	0.36
>35	4	1,410	0.98 (0.72–1.33)	34%	0.88

CI, confidence interval; MD, mean difference; OR, odds ratio; GnRH, gonadotrophin releasing hormone; GnRH-A, GnRH, antagonist; GnRH-a, GnRH, agonist; NA, not available.

**FIGURE 7:**
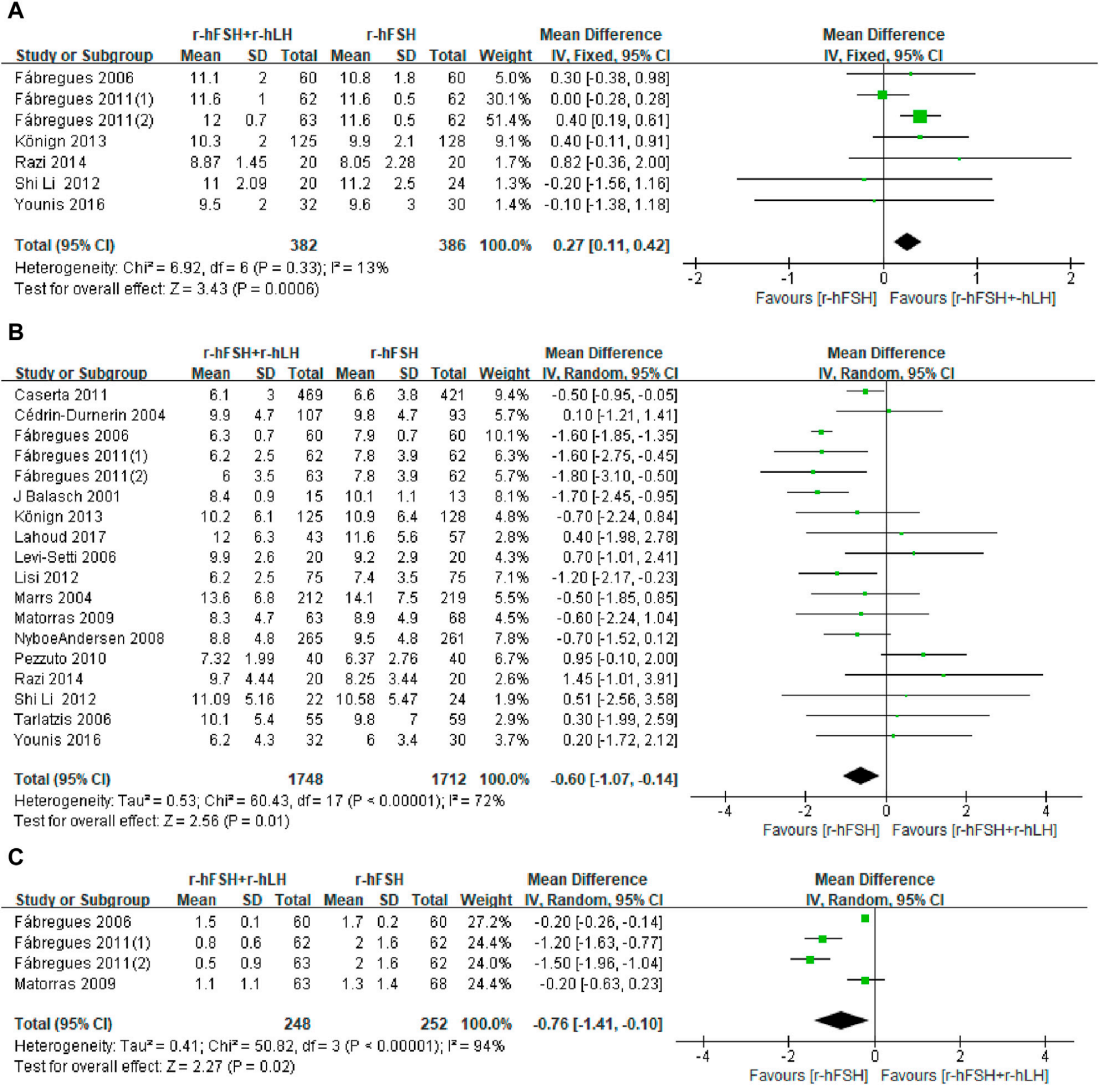
Forest plots of the outcomes. Recombinant human follicle-stimulating hormone (r-hFSH)/recombinant human luteinizing hormone (r-hLH) co-treatment *versus* r-hFSH treatment: endometrial thickness (EMT) on trigger day **(A)**, number of oocytes retrieved **(B)** and number of high-quality embryos **(C)**. The studies are listed by the first author’s last name followed by the year of publication. CI, confidence interval; r-hFSH, recombinant human follicle-stimulating hormone; r-hLH, recombinant human luteinizing hormone.

**EMT on trigger day.** EMT was investigated in seven RCTs (Table 1). The study by Vuong et al. (2015) used the median and interquartile range for the statistical description of continuous variables, which could not be used for meta-analysis, where the addition of r-hLH did not significantly increase EMT. The pooled results from the remaining six studies showed that the r-hFSH/r-hLH co-treatment significantly increased EMT on trigger day compared to r-hFSH alone (MD = 0.27; 95% CI: 0.11–0.42; P = 0.0006, Figure 7). The QoE was high (Table 2). The subgroup analysis indicated a better effect on improving EMT by the GnRH-a long protocol than the GnRH-A protocol. Moreover, with a dosage of r-hLH of 75 IU/day, EMT on trigger day was significantly thicker. In addition, among patients ≤35 years old, adding r-hLH had a more significant effect on improving EMT than those >35 years old (Table 4).

**Number of oocytes retrieved.** Of the twenty-one studies reporting the number of oocytes retrieved, four could not be meta-analyzed because the data required for statistical analysis was not adequately reported (Humaidan et al., 2004; Hong et al., 2012; Musters et al., 2012; Vuong et al., 2015). However, the results of these four studies on the effect of r-hLH supplementation on the number of oocytes retrieved were consistent, that is, the number of oocytes retrieved did not increase with the supplementation of r-hLH. Meta-analysis also showed a similar outcome that the r-hFSH/r-hLH co-treatment showed no improvement to the number of oocytes retrieved in women undergoing IVF/ICSI (MD = −0.60; 95% CI: −1.07 to −0.14; *I* ^*2*^ = 72%; p = 0.01) (Figure 7). The QoE was low due to publication bias and significant inconsistency with unexplained heterogeneity (Table 2). The subgroup analysis showed worse outcomes with the GnRH-a long protocol than the GnRH-A protocol. Notably, r-hLH at 150 IU/day or 37.5 IU/day reduced oocyte numbers. The effect of r-hLH supplementation was less potent among patients ≤35 years old than those >35 years old (Table 4).

**FIGURE 8:**
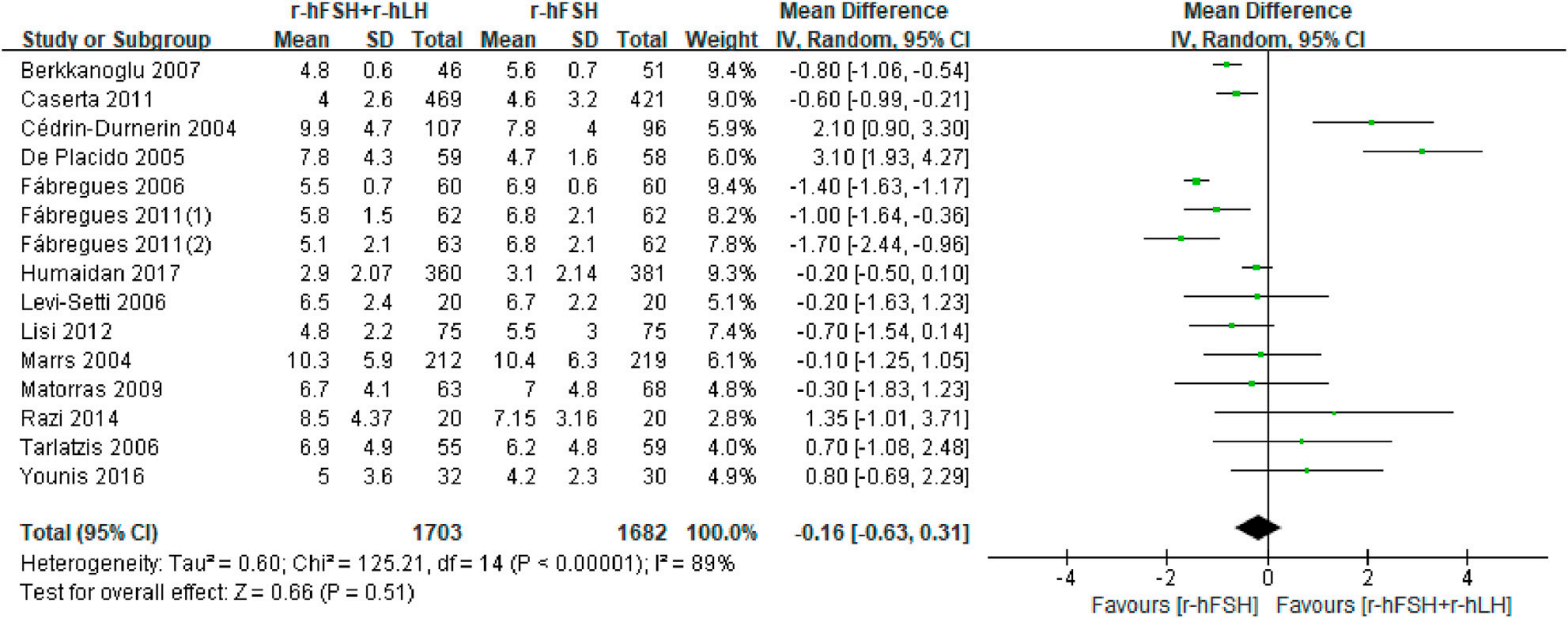
Forest plot of number of MII oocytes retrieved. r-hFSH/r-hLH co-treatment (r-hFSH + r-hLH) *versus* r-hFSH treatment (r-hFSH).

**Number of MII oocytes retrieved.** Fifteen studies with 3,323 participants were included in the analysis (Table 1). Balasch et al. (2001) reported the proportion of oocytes at MII but not the absolute number, which was excluded from the meta-analysis. This study showed that the addition of r-hLH significantly reduced the proportion of MII oocytes (74% *versus* 84%, P< 0.05). The study by Hong et al. (2012) was also excluded due to the use of median and interquartile intervals to describe the number of MII oocytes, which showed no effect. Overall, the results of meta-analysis echoed the non-significant effect on the number of MII oocytes retrieved (MD = −0.16, 95% CI: −0.63 to 0.31; P= 0.51; *I* ^*2*^ = 89%) (Figure 8). The studies had a low QoE (Table 2). Results from subgroup analysis showed a significant reduction in MII oocytes retrieved with a microflare stimulation protocol or an r-hLH dose of 37.5 IU/day compared with the use of r-hFSH alone. In addition, the effect of r-hLH was more potent among patients >35 years old than ≤35 years old (Table 7).

**TABLE 7:** Effect estimate and heterogeneity of subgroup analysis for number of MII oocytes retrieved.

Subgroup	No. of studies (n)	No. of women (n)	Effect estimate OR/MD (95% CI)	*I* ^*2*^	P
Ovarian stimulation protocol					
GnRH-a long protocol	10	2,573	−0.30 (−0.88 to 0.28)	90%	0.32
GnRH-A protocol	3	305	0.95 (−0.42–2.32)	67%	0.18
Microflare stimulation protocol**	1	97	−0.80 (−1.06 to −0.54)	NA	≤0.00001
r-hLH dosage					
75 IU/day	8	1768	−0.30 (−0.89 to 0.28)	80%	0.31
150 IU/day	5	1,083	0.13 (−0.95–1.21)	95%	0.81
37.5 IU/day*	1	125	−1.00 (−1.64 to −0.36)	NA	0.002
keep the ratio of FSH:LH to 2:1	1	62	0.80 (−0.69–2.29)	NA	0.29
Average age of participants in both groups					
≤35	9	2,378	0.46 (−0.24–1.16)	85%	0.20
>35**	5	597	−1.00 (−1.44 to −0.55)	77%	<0.0001

CI, confidence interval; MD, mean difference; OR, odds ratio; GnRH, gonadotrophin releasing hormone; GnRH-A, GnRH, antagonist; GnRH-a, GnRH, agonist; r-hFSH, recombinant human follicle-stimulating hormone; r-hLH, recombinant human luteinizing hormone; NA, not available; *, P< 0.05; **, P< 0.001.

**Number of high-quality embryos.** Only three (Fábregues et al., 2006; Matorras et al., 2009; Fábregues et al., 2011) out of five studies reporting the number of high-quality embryos (Table 1) and had sufficient data for meta-analysis. Two other studies (Musters et al., 2012; Vuong et al., 2015) showed no effect of r-hFSH/r-hLH on the number of high-quality embryos. Nevertheless, the pooled results showed that the addition of r-hLH did not show a beneficial effect on the number of high-quality embryos either (MD = −0.76, 95% CI: −1.41 to −0.10; P= 0.02; *I* ^*2*^ = 94%) (Figure 7). The QoE was low due to significant inconsistency with unexplained heterogeneity and imprecision with small studies and/or wide confidence intervals (Table 2).

**High-quality embryo rate.** Four studies evaluated high-quality embryo rates (Table 1). However, there was no sufficient sample size for meta-analysis. One study (Lisi et al., 2012) showed that r-hFSH/r-hLH significantly increased the rate of high-quality embryos (95.7% *versus* 91.6%, p < 0.01), whereas the other three studies showed that the r-hFSH/r-hLH co-treatment had no significant effect on the high-quality embryo rate compared with the r-hFSH treatment.

### 3.4 Sensitivity analysis and publication bias

Most of the results were stable and remained unchanged after sensitivity analysis, except for the results on the number of MII oocytes retrieved. The Egger’s test and funnel plot were used to test for potential publication bias. As shown in Supplementary Figure S1 and Supplementary Figure S2, no asymmetry was found in the funnel plot of EMT on trigger day and the number of high-quality embryos, which is further supported by Egger’s tests (*P*_*EMT*_ = 0.848, Supplementary Figure S3; *P*_*number of high-quality embryos*_ = 0.192; Supplementary Figure S4). Nevertheless, as presented in Supplementary Figure S5, the funnel plot of the number of oocytes retrieved suggests the existence of publication bias, which is further confirmed by Egger’s test (*P*_*number of oocytes retrieved*_ = 0.04, Supplementary Figure S6). The presence of publication bias may affect the reliability of the results, which needs to be interpreted with caution.

## 4 Discussion

This literature review and meta-analysis comprehensively evaluated the effect of r-hFSH/r-hLH co-treatment *versus* r-hFSH alone on EMT during COS in women undergoing IVF/ICSI-ET, which has added new evidence to the literature. We found that r-hLH addition to r-hFSH significantly improved EMT on trigger day. Furthermore, this study took the initiative to assess the effects of different dose regimes of r-hLH, which is normally adjusted based on patient’s follicle development and hormone levels. Our meta-analysis results show that r-hLH of 75 IU/day was beneficial in optimizing conception success.

Low endogenous LH levels certainly have a negative impact on the outcome of ART (Luo et al., 2022). Compared with the previous systematic reviews and meta-analyses comparing r-hFSH/r-hLH co-treatment *versus* r-hFSH alone, this study is the first to include several outcome measures that are of interest to both clinicians and patients, including oocytes, embryos, EMT and IVF outcomes. The most striking result in the current study was that adding r-hLH can significantly increase EMT on trigger day. During the menstrual cycle, endometrium proliferates mainly under the influence of estrogen (Deligdisch-Schor and Miceli, 2020). According to the “two-cell, two-gonadotropin” theory, LH supplementation may increase the androgen level that may be converted to estrogen (Papaleo et al., 2014). Thus, the addition of r-hLH during ovarian stimulation may increase EMT through increasing endogenous estrogen levels.

To date, evidence supports the idea that adding LH improves ART outcomes (Conforti et al., 2019). Notely, in this meta-analysis, the combination of r-hFSH and r-hLH did not improve the number of oocytes retrieved, MII oocytes retrieved and high-quality embryos compared with r-hFSH alone (Table 2). Although EMT on trigger day was increased by this treatment, the IVF outcomes, including IR, LBR, CPR, and MR, seem to be unaffected (Table 2). A retrospective cohort study comprising 1565 COS cycles showed that supplementing with LH can increase the success rate of conception and live birth (Paterson et al., 2012). A systematic review and meta-analysis of the effects of r-hLH supplementation in women with low ovarian stimulation response showed that IR and CPR were higher with r-hFSH/r-hLH dual treatment compared with r-hFSH monotherapy (Conforti et al., 2019). However, our study did not show a beneficial effect of r-hFSH/r-hLH dual treatment on IVF outcomes. This may be due to the population in our study, which included both normal and low responders.

Pregnancy outcomes are influenced by both the embryo and the endometrium qualities. The embryo also plays an important role in IVF outcomes. In our study, the addition of r-hLH did not improve the number of embryos, but adversely affected oocyte retrieval and formation of high-quality embryos (Table 2). The results suggested that r-hLH supplementation during ovarian stimulation did not improve oocyte retrieval, maturation of oocytes and embryo quality. Adding r-hLH can reduce oocyte retrieval but does not significantly affect the number of mature oocytes. This may be because LH can inhibit small pre-ovulation follicles and thus facilitate the maturation of dominant follicles during COS (Filicori et al., 2001). A multicenter retrospective study suggested that r-hLH supplementation could improve embryo quality in GnRH-A protocol (Wang et al., 2022), which is inconsistent with our findings. In our meta-analysis, fewer high-quality embryos are likely affected by the lower number of oocytes retrieved. Moreover, since only four studies reported the effect of r-hFSH/r-hLH co-treatment on the number of high-quality embryos, subgroup analysis could not be performed. Moreover, all four studies used GnRH-a long protocol for ovarian stimulation. Therefore, further research is required to investigate the precise effect of r-hLH addition on embryo quality using other protocols. Since r-hFSH/r-hLH significantly improved EMT quality, it is reasonable to postulate that the positive effect of r-hLH on endometrium compensated for its adverse effect on oocytes and embryos, resulting in an unchanged final IVF outcome. A 2017 Cochrane review investigated the impact of the combination of r-hFSH/r-hLH co-treatment on the live birth rate (Mochtar et al., 2017). Although the review found no impact of such an approach on the live birth rate, the evidence quality is low. However, this review did not examine the other parameters that determine the pregnancy outcome (Mochtar et al., 2017). Our study complemented this previous review and provided the reasons leading to such outcomes.

In the subgroup analysis based on the COS protocol, r-hLH significantly increased EMT in the GnRH-a long protocol, which was not found in the GnRH-A protocol (Table 4). This is consistent with a systematic review and meta-analysis of GnRH-a long protocol *versus* GnRH-A protocol on EMT in women with polycystic ovary syndrome (Kadoura et al., 2022). Homeobox A10, a marker of endometrial receptivity, plays an important role in endometrial proliferation, which is lower in the endometrium during the GnRH-A cycle compared to the GnRH-a cycle (Chen et al., 2020). In addition, other endometrial receptivity markers, such as leukemia-inhibitory factor and integrin β3, were increased by the GnRH-a protocol compared to the GnRH-A protocol (Ruan et al., 2006). The current meta-analysis included both GnRH-a and GnRH-A protocols, which may compromise the effect on CPR and LBR due to inadequate endometrial receptivity markers by GnRH-A protocol.

According to the “LH window” theory, either too high or too low LH levels can adversely affect follicle development (Balasch and Fábregues, 2002). Insufficient LH levels can impair estrogen synthesis, affecting follicle maturation and the growth of the corpus luteum. On the other hand, high LH levels can inhibit granular cell division and the premature start of follicular meiosis, eventually leading to follicular atresia and luteinization. Mous experiments have shown that continuous administration of a fixed dose of LH during IVF treatment can directly inhibit tissue regeneration in endometrial stem cells (Park et al., 2022), suggesting an optimal dose of exogenous LH is required to prevent any adverse effects on the endometrium. Indeed, Either too high or too low LH supplementation can lead to poor endometrial hyperplasia and affect IVF outcomes (Shoham, 2002). The current meta-analysis showed that an r-hLH dose of 75 IU/day can significantly increase EMT and CPR (Table 4). Such benefits were not observed with 37.5 IU/day or 150 IU/day, which were actually detrimental to oocyte retrieval and oocyte maturation (Table 4; Table 7).

The strength included the decent number of studies included, which makes the conclusion more robust. This study was the first to evaluate the dose effect of the r-hLH regime on clinical outcomes of IVF/ICSI-ET. Compared with previous studies, our study was more comprehensive by including the oocyte, embryo, endometrium and IVF outcomes. A subgroup analysis on the efficacy of r-hLH was performed according to ovarian stimulation protocols, which further investigated the benefit of additional r-hLH on EMT. We also need to acknowledge the limitations. Only EMT was evaluated, which is a knowledge gap in previous studies that failed to address the type of endometrium or endometrial function. Additionally, we cannot correct potential sampling bias in the original studies.

## 5 Conclusion

The r-hFSH and r-hLH co-treatment during COS in women undergoing IVF/ICSI-ET can significantly improve EMT on trigger day compared with r-hFSH alone. An r-hLH dose of 75 IU/day may be considered for optimal pregnancy outcomes, which still require further clinical studies to support this dosing regime.

## Data Availability

The original contributions presented in the study are included in the article/Supplementary Material, further inquiries can be directed to the corresponding authors.
