# Supplementary material for: Recombinant human luteinizing hormone increases endometrial thickness in women undergoing assisted fertility treatments: a systematic review and meta-analysis
Source: Front Pharmacol. 2024 Jul 29;15:1434625. doi: 10.3389/fphar.2024.1434625 (PMC11317380; doi:10.3389/fphar.2024.1434625)
Supplement: Supplementary file 1 [file DataSheet1.docx]

Supplementary Material

Recombinant human luteinizing hormone increases endometrial thickness in women undergoing assisted fertility treatments: a systematic review and meta-analysis

**Routong Mao^1^,** **Xiaohong Hou^1^,** **Xiao Feng^1^, Ruina Wang^1^, Xiaofan Fei^1^, Junzhao Zhao^1^, Hui Chen ^2^** †**, Jing Cheng** **^1^** †*

† These authors share senior authorship

^1^Reproductive Center, Department of Obstetrics and Gynecology, The Second Affiliated Hospital and Yuying Children’s Hospital of Wenzhou Medical University, 306 Hualong Qiao Road, Lucheng District, Wenzhou 325088, Zhejiang Province, China

^2^ School of Life Sciences, Faculty of Science, University of Technology Sydney, 15 Broadway, Ultimo 2007, New South Wales, Australia

**Supplemental Table 1** Prisma checklist

| **Section and Topic** | **Item #** | **Checklist item** | **Location where item is reported** |
| --- | --- | --- | --- |
| **TITLE** | | |  |
| Title | 1 | Identify the report as a systematic review. | **• Title.** |
| **ABSTRACT** | | |  |
| Abstract | 2 | See the PRISMA 2020 for Abstracts checklist. | **• Abstract.** |
| **INTRODUCTION** | | |  |
| Rationale | 3 | Describe the rationale for the review in the context of existing knowledge. | **• Introduction.** |
| Objectives | 4 | Provide an explicit statement of the objective(s) or question(s) the review addresses. | **• Introduction.** |
| **METHODS** | | |  |
| Eligibility criteria | 5 | Specify the inclusion and exclusion criteria for the review and how studies were grouped for the syntheses. | **• Inclusion and exclusion criteria.** |
| Information sources | 6 | Specify all databases, registers, websites, organisations, reference lists and other sources searched or consulted to identify studies. Specify the date when each source was last searched or consulted. | **• Search strategy.** |
| Search strategy | 7 | Present the full search strategies for all databases, registers and websites, including any filters and limits used. | **• Supplementary Table 2.** |
| Selection process | 8 | Specify the methods used to decide whether a study met the inclusion criteria of the review, including how many reviewers screened each record and each report retrieved, whether they worked independently, and if applicable, details of automation tools used in the process. | **• Selection process and data extraction.** |
| Data collection process | 9 | Specify the methods used to collect data from reports, including how many reviewers collected data from each report, whether they worked independently, any processes for obtaining or confirming data from study investigators, and if applicable, details of automation tools used in the process. | **•Selection process and data extraction .** |
| Data items | 10a | List and define all outcomes for which data were sought. Specify whether all results that were compatible with each outcome domain in each study were sought (e.g. for all measures, time points, analyses), and if not, the methods used to decide which results to collect. | **•Selection process and data extraction .** |
|  | 10b | List and define all other variables for which data were sought (e.g. participant and intervention characteristics, funding sources). Describe any assumptions made about any missing or unclear information. | **•Selection process and data extraction .** |
| Study risk of bias assessment | 11 | Specify the methods used to assess risk of bias in the included studies, including details of the tool(s) used, how many reviewers assessed each study and whether they worked independently, and if applicable, details of automation tools used in the process. | **• Assessment of risk of bias in included studies.** |
| Effect measures | 12 | Specify for each outcome the effect measure(s) (e.g. risk ratio, mean difference) used in the synthesis or presentation of results. | **• Statistical analysis.** |
| Synthesis methods | 13a | Describe the processes used to decide which studies were eligible for each synthesis (e.g. tabulating the study intervention characteristics and comparing against the planned groups for each synthesis (item #5)). | **• Statistical analysis.** |
|  | 13b | Describe any methods required to prepare the data for presentation or synthesis, such as handling of missing summary statistics, or data conversions. | **•Selection process and data extraction .** |
|  | 13c | Describe any methods used to tabulate or visually display results of individual studies and syntheses. | **• Statistical analysis.** |
|  | 13d | Describe any methods used to synthesize results and provide a rationale for the choice(s). If meta-analysis was performed, describe the model(s), method(s) to identify the presence and extent of statistical heterogeneity, and software package(s) used. | **• Statistical analysis.** |
|  | 13e | Describe any methods used to explore possible causes of heterogeneity among study results (e.g. subgroup analysis, meta-regression). | **• Subgroup and sensitivity analysis.** |
|  | 13f | Describe any sensitivity analyses conducted to assess robustness of the synthesized results. | **• Subgroup and sensitivity analysis.** |
| Reporting bias assessment | 14 | Describe any methods used to assess risk of bias due to missing results in a synthesis (arising from reporting biases). | **• Publication bias assessment.** |
| Certainty assessment | 15 | Describe any methods used to assess certainty (or confidence) in the body of evidence for an outcome. | **• Assessment of certainty of evidence.** |
| **RESULTS** | | |  |
| Study selection | 16a | Describe the results of the search and selection process, from the number of records identified in the search to the number of studies included in the review, ideally using a flow diagram. | **• Study characteristics.**  **• Figure 1. Flowchart of study design.** |
|  | 16b | Cite studies that might appear to meet the inclusion criteria, but which were excluded, and explain why they were excluded. | **• Study characteristics.** |
| Study characteristics | 17 | Cite each included study and present its characteristics. | **• Study characteristics.** |
| Risk of bias in studies | 18 | Present assessments of risk of bias for each included study. | **• Risk of bias.**  **• Figure 2. Risk of bias assessment of included studies.** |
| Results of individual studies | 19 | For all outcomes, present, for each study: (a) summary statistics for each group (where appropriate) and (b) an effect estimate and its precision (e.g. confidence/credible interval), ideally using structured tables or plots. | **• A forest plot was formed to summarize the results of included studies for each outcome and put in the “Results” section.** |
| Results of syntheses | 20a | For each synthesis, briefly summarise the characteristics and risk of bias among contributing studies. | **• This was reported at an outcome-level. Each outcome was discussed separately in the “Results” section in an individual paragraph, and the results were summarized in the forest plots.** |
|  | 20b | Present results of all statistical syntheses conducted. If meta-analysis was done, present for each the summary estimate and its precision (e.g. confidence/credible interval) and measures of statistical heterogeneity. If comparing groups, describe the direction of the effect. | **• This was reported at an outcome-level. Each outcome was discussed separately in the “Results” section in an individual paragraph, and the results were summarized in the forest plots.** |
|  | 20c | Present results of all investigations of possible causes of heterogeneity among study results. | **• This was reported at an outcome-level (when necessary). Each outcome was discussed separately in the “Results” section in an individual paragraph.** |
|  | 20d | Present results of all sensitivity analyses conducted to assess the robustness of the synthesized results. | **• This was reported at an outcome-level (when necessary). Each**  **outcome was discussed separately in the “Results” section in an individual paragraph.** |
| Reporting biases | 21 | Present assessments of risk of bias due to missing results (arising from reporting biases) for each synthesis assessed. | **• Sensitivity analysis and publication bias.** |
| Certainty of evidence | 22 | Present assessments of certainty (or confidence) in the body of evidence for each outcome assessed. | **• This was reported at an outcome-level. Each outcome was**  **discussed separately in the “Results” section in an individual paragraph.**  **•** **Table 2. Clinical outcomes.** |
| **DISCUSSION** | | |  |
| Discussion | 23a | Provide a general interpretation of the results in the context of other evidence. | **• Discussion.** |
|  | 23b | Discuss any limitations of the evidence included in the review. | **• Discussion.** |
|  | 23c | Discuss any limitations of the review processes used. | **• Discussion.** |
|  | 23d | Discuss implications of the results for practice, policy, and future research. | **• Conclusions.** |
| **OTHER INFORMATION** | | |  |
| Registration and protocol | 24a | Provide registration information for the review, including register name and registration number, or state that the review was not registered. | **• Protocol and registration, PROSPERO (CRD42023454584).** |
|  | 24b | Indicate where the review protocol can be accessed, or state that a protocol was not prepared. | **• Protocol and registration, PROSPERO (CRD42023454584).** |
|  | 24c | Describe and explain any amendments to information provided at registration or in the protocol. | **• Protocol and registration, PROSPERO (CRD42023454584).** |
| Support | 25 | Describe sources of financial or non-financial support for the review, and the role of the funders or sponsors in the review. | **• Funding.** |
| Competing interests | 26 | Declare any competing interests of review authors. | **• Conflicts of interest.** |
| Availability of data, code and other materials | 27 | Report which of the following are publicly available and where they can be found: template data collection forms; data extracted from included studies; data used for all analyses; analytic code; any other materials used in the review. | **•** **Data availability.** |

**Supplemental Table 2** Search strategy of English databases

**PubMed**

| 14 | (((("Reproductive Techniques, Assisted"[Mesh]) OR (((((((((((((((((Assisted Reproductive Technique) OR (Reproductive Technique, Assisted)) OR (Technique, Assisted Reproductive)) OR (Techniques, Assisted Reproductive)) OR (Assisted Reproductive Technics)) OR (Assisted Reproductive Technic)) OR (Reproductive Technic, Assisted)) OR (Reproductive Technics, Assisted)) OR (Technic, Assisted Reproductive)) OR (Technics, Assisted Reproductive)) OR (Assisted Reproductive Techniques)) OR (Reproductive Technology, Assisted)) OR (Assisted Reproductive Technologies)) OR (Assisted Reproductive Technology)) OR (Reproductive Technologies, Assisted)) OR (Technologies, Assisted Reproductive)) OR (Technology, Assisted Reproductive))) OR (("Fertilization in Vitro"[Mesh]) OR ((((((((((((((Fertilization in Vitro) OR (In Vitro Fertilization)) OR (In Vitro Fertilizations)) OR (Test-Tube Fertilization)) OR (Fertilization, Test-Tube)) OR (Fertilizations, Test-Tube)) OR (Test Tube Fertilization)) OR (Test-Tube Fertilizations)) OR (Fertilizations in Vitro)) OR (Test-Tube Babies)) OR (Babies, Test-Tube)) OR (Baby, Test-Tube)) OR (Test Tube Babies)) OR (Test-Tube Baby)))) AND (("Luteinizing Hormone"[Mesh]) OR (((((((((Hormone, Luteinizing) OR (Lutropin)) OR (LH (Luteinizing Hormone))) OR (ICSH (Interstitial Cell Stimulating Hormone))) OR (Interstitial Cell-Stimulating Hormone)) OR (Hormone, Interstitial Cell-Stimulating)) OR (Interstitial Cell Stimulating Hormone)) OR (Luteozyman)) OR (Luteoziman)))) AND (("Randomized Controlled Trial" [Publication Type]) OR (((((((((((Controlled clinical trial) OR (Random allocation)) OR (Double-blind)) OR (Single-blind)) OR (Placebo)) OR (Randomly)) OR (Randomized)) OR (Clinical trial)) OR (Trial)) OR (RCT)) OR (Random))) | 1,003 |
| --- | --- | --- |
| 13 | (("Randomized Controlled Trial" [Publication Type])) OR (((((((((((Controlled clinical trial) OR (Random allocation)) OR (Double-blind)) OR (Single-blind)) OR (Placebo)) OR (Randomly)) OR (Randomized)) OR (Clinical trial)) OR (Trial)) OR (RCT)) OR (Random)) | 2,830,657 |
| 12 | ((((((((((Controlled clinical trial) OR (Random allocation)) OR (Double-blind)) OR (Single-blind)) OR (Placebo)) OR (Randomly)) OR (Randomized)) OR (Clinical trial)) OR (Trial)) OR (RCT)) OR (Random) | 2,830,657 |
| 11 | ("Randomized Controlled Trial" [Publication Type]) | 603,656 |
| 10 | ("Luteinizing Hormone"[Mesh]) OR (((((((((Hormone, Luteinizing) OR (Lutropin)) OR (LH (Luteinizing Hormone))) OR (ICSH (Interstitial Cell Stimulating Hormone))) OR (Interstitial Cell-Stimulating Hormone)) OR (Hormone, Interstitial Cell-Stimulating)) OR (Interstitial Cell Stimulating Hormone)) OR (Luteozyman)) OR (Luteoziman)) | 63,341 |
| 9 | ((((((((Hormone, Luteinizing) OR (Lutropin)) OR (LH (Luteinizing Hormone))) OR (ICSH (Interstitial Cell Stimulating Hormone))) OR (Interstitial Cell-Stimulating Hormone)) OR (Hormone, Interstitial Cell-Stimulating)) OR (Interstitial Cell Stimulating Hormone)) OR (Luteozyman)) OR (Luteoziman) | 63,341 |
| 8 | "Luteinizing Hormone"[Mesh] | 48,192 |
| 7 | (("Reproductive Techniques, Assisted"[Mesh]) OR (((((((((((((((((Assisted Reproductive Technique) OR (Reproductive Technique, Assisted)) OR (Technique, Assisted Reproductive)) OR (Techniques, Assisted Reproductive)) OR (Assisted Reproductive Technics)) OR (Assisted Reproductive Technic)) OR (Reproductive Technic, Assisted)) OR (Reproductive Technics, Assisted)) OR (Technic, Assisted Reproductive)) OR (Technics, Assisted Reproductive)) OR (Assisted Reproductive Techniques)) OR (Reproductive Technology, Assisted)) OR (Assisted Reproductive Technologies)) OR (Assisted Reproductive Technology)) OR (Reproductive Technologies, Assisted)) OR (Technologies, Assisted Reproductive)) OR (Technology, Assisted Reproductive))) OR (("Fertilization in Vitro"[Mesh]) OR ((((((((((((((Fertilization in Vitro) OR (In Vitro Fertilization)) OR (In Vitro Fertilizations)) OR (Test-Tube Fertilization)) OR (Fertilization, Test-Tube)) OR (Fertilizations, Test-Tube)) OR (Test Tube Fertilization)) OR (Test-Tube Fertilizations)) OR (Fertilizations in Vitro)) OR (Test-Tube Babies)) OR (Babies, Test-Tube)) OR (Baby, Test-Tube)) OR (Test Tube Babies)) OR (Test-Tube Baby))) | 98,648 |
| 6 | ("Fertilization in Vitro"[Mesh]) OR ((((((((((((((Fertilization in Vitro) OR (In Vitro Fertilization)) OR (In Vitro Fertilizations)) OR (Test-Tube Fertilization)) OR (Fertilization, Test-Tube)) OR (Fertilizations, Test-Tube)) OR (Test Tube Fertilization)) OR (Test-Tube Fertilizations)) OR (Fertilizations in Vitro)) OR (Test-Tube Babies)) OR (Babies, Test-Tube)) OR (Baby, Test-Tube)) OR (Test Tube Babies)) OR (Test-Tube Baby)) | 55,827 |
| 5 | "Fertilization in Vitro"[Mesh] | 40,941 |
| 4 | (((((((((((((Fertilization in Vitro) OR (In Vitro Fertilization)) OR (In Vitro Fertilizations)) OR (Test-Tube Fertilization)) OR (Fertilization, Test-Tube)) OR (Fertilizations, Test-Tube)) OR (Test Tube Fertilization)) OR (Test-Tube Fertilizations)) OR (Fertilizations in Vitro)) OR (Test-Tube Babies)) OR (Babies, Test-Tube)) OR (Baby, Test-Tube)) OR (Test Tube Babies)) OR (Test-Tube Baby) | 55,827 |
| 3 | ("Reproductive Techniques, Assisted"[Mesh]) OR (((((((((((((((((Assisted Reproductive Technique) OR (Reproductive Technique, Assisted)) OR (Technique, Assisted Reproductive)) OR (Techniques, Assisted Reproductive)) OR (Assisted Reproductive Technics)) OR (Assisted Reproductive Technic)) OR (Reproductive Technic, Assisted)) OR (Reproductive Technics, Assisted)) OR (Technic, Assisted Reproductive)) OR (Technics, Assisted Reproductive)) OR (Assisted Reproductive Techniques)) OR (Reproductive Technology, Assisted)) OR (Assisted Reproductive Technologies)) OR (Assisted Reproductive Technology)) OR (Reproductive Technologies, Assisted)) OR (Technologies, Assisted Reproductive)) OR (Technology, Assisted Reproductive)) | 87,573 |
| 2 | ((((((((((((((((Assisted Reproductive Technique) OR (Reproductive Technique, Assisted)) OR (Technique, Assisted Reproductive)) OR (Techniques, Assisted Reproductive)) OR (Assisted Reproductive Technics)) OR (Assisted Reproductive Technic)) OR (Reproductive Technic, Assisted)) OR (Reproductive Technics, Assisted)) OR (Technic, Assisted Reproductive)) OR (Technics, Assisted Reproductive)) OR (Assisted Reproductive Techniques)) OR (Reproductive Technology, Assisted)) OR (Assisted Reproductive Technologies)) OR (Assisted Reproductive Technology)) OR (Reproductive Technologies, Assisted)) OR (Technologies, Assisted Reproductive)) OR (Technology, Assisted Reproductive) | 87,573 |
| 1 | "Reproductive Techniques, Assisted"[Mesh] | 79,992 |

**Cochrane Library**

| #1 | MeSH descriptor: [Luteinizing Hormone] explode all trees | 1,762 |
| --- | --- | --- |
| #2 | (Hormone, Luteinizing or Lutropin or LH (Luteinizing Hormone) or ICSH (Interstitial Cell Stimulating Hormone) or Interstitial Cell-Stimulating Hormone or Hormone, Interstitial Cell-Stimulating or Interstitial Cell Stimulating Hormone or Luteozyman or Luteoziman):ti,ab,kw (Word variations have been searched) | 4,433 |
| #3 | #1 or #2 | 4,441 |
| #4 | MeSH descriptor: [Fertilization in Vitro] explode all trees | 2,618 |
| #5 | (In Vitro Fertilization or In Vitro Fertilizations or Test-Tube Fertilization or Fertilization, Test-Tube or Fertilizations, Test-Tube or Test Tube Fertilization or Test-Tube Fertilizations or Fertilizations in Vitro or Test-Tube Babies or Babies, Test-Tube or Baby, Test-Tube or Test Tube Babies or Test-Tube Baby):ti,ab,kw (Word variations have been searched) | 5,804 |
| #6 | #4 or #5 | 6,027 |
| #7 | MeSH descriptor: [Reproductive Techniques, Assisted] explode all trees | 4,465 |
| #8 | (Assisted Reproductive Technique or Reproductive Technique, Assisted or Technique, Assisted Reproductive or Techniques, Assisted Reproductive or Assisted Reproductive Technics or Assisted Reproductive Technic or Reproductive Technic, Assisted or Reproductive Technics, Assisted or Technic, Assisted Reproductive or Technics, Assisted Reproductive or Assisted Reproductive Techniques or Reproductive Technology, Assisted or Assisted Reproductive Technologies or Assisted Reproductive Technology or Reproductive Technologies, Assisted or Technologies, Assisted Reproductive or Technology, Assisted Reproductive):ti,ab,kw (Word variations have been searched) | 1,628 |
| #9 | #7 or #8 | 5,572 |
| #10 | MeSH descriptor: [Randomized Controlled Trial] explode all trees | 25,732 |
| #11 | (Controlled clinical trial or Random allocation or Double-blind or Single-blind or Placebo or Randomly or Randomized or Clinical trial or Trial or RCT or Random):ti,ab,kw (Word variations have been searched) | 1,496,453 |
| #12 | #10 or #11 | 1,496,454 |
| #13 | #3 and #6 and #9 and #12 | 376 |

**Web of science**

| 1 | TS=(Luteinizing Hormone OR Hormone, Luteinizing OR Lutropin OR LH (Luteinizing Hormone) OR ICSH (Interstitial Cell Stimulating Hormone) OR Interstitial Cell-Stimulating Hormone OR Hormone, Interstitial Cell-Stimulating OR Interstitial Cell Stimulating Hormone OR Luteozyman OR Luteoziman) and Preprint Citation Index (Exclude – Database) | 88,147 |
| --- | --- | --- |
| 2 | TS=(Fertilization in Vitro OR In Vitro Fertilization OR In Vitro Fertilizations OR Test-Tube Fertilization OR Fertilization, Test-Tube OR Fertilizations, Test-Tube OR Test Tube Fertilization OR Test-Tube Fertilizations OR Fertilizations in Vitro OR Test-Tube Babies OR Babies, Test-Tube OR Baby, Test-Tube OR Test Tube Babies OR Test-Tube Baby OR Reproductive Techniques, Assisted OR Assisted Reproductive Technique OR Reproductive Technique, Assisted OR Technique, Assisted Reproductive OR Techniques, Assisted Reproductive OR Assisted Reproductive Technics OR Assisted Reproductive Technic OR Reproductive Technic, Assisted OR Reproductive Technics, Assisted OR Technic, Assisted Reproductive OR Technics, Assisted Reproductive OR Assisted Reproductive Techniques OR Reproductive Technology, Assisted OR Assisted Reproductive Technologies OR Assisted Reproductive Technology OR Reproductive Technologies, Assisted OR Technologies, Assisted Reproductive OR Technology, Assisted Reproductive) and Preprint Citation Index (Exclude – Database) | 127,469 |
| 3 | TS=(Randomized Controlled Trial OR Controlled clinical trial OR Random allocation OR Double-blind OR Single-blind OR Placebo OR Randomly OR Randomized OR Clinical trial OR Trial OR RCT OR Random) and Preprint Citation Index (Exclude – Database) | 4,252,369 |
| 4 | #1 AND #2 AND #3 and Preprint Citation Index (Exclude – Database) | 1,039 |

**EMBASE**

| #59. | #11 AND #26 AND #45 AND #58 | 194 |
| --- | --- | --- |
| #58. | #46 OR #47 OR #48 OR #49 OR #50 OR #51 OR #52 OR #53 OR #54 OR #55 OR #56 OR #57 | 3,623,783 |
| #57. | random | 442,180 |
| #56. | rct | 59,816 |
| #55. | trial | 2,603,907 |
| #54. | clinical AND trial | 2,314,723 |
| #53. | randomized | 1,401,324 |
| #52. | randomly | 546,297 |
| #51. | placebo | 529,024 |
| #50. | 'single blind' | 59,869 |
| #49. | 'double blind' | 292,024 |
| #48. | random AND allocation | 6,597 |
| #47. | controlled AND clinical AND trial | 1,438,355 |
| #46. | randomized AND controlled AND trial | 1,113,952 |
| #45. | #27 OR #28 OR #29 OR #30 OR #31 OR #32 OR #33 OR #34 OR #35 OR #36 OR #37 OR #38 OR #39 OR #40 OR #41 OR #42 OR #43 OR #44 | 26,886 |
| #44. | technology, AND assisted AND reproductive | 15,971 |
| #43. | technologies, AND assisted AND reproductive | 7,526 |
| #42. | reproductive AND technologies, AND assisted | 7,526 |
| #41. | assisted AND reproductive AND technology | 15,971 |
| #40. | assisted AND reproductive AND technologies | 7,526 |
| #39. | reproductive AND technology, AND assisted | 15,971 |
| #38. | assisted AND reproductive AND techniques | 6,811 |
| #37. | technics, AND assisted AND reproductive | 37 |
| #36. | technic, AND assisted AND reproductive | 8 |
| #35. | reproductive AND technics, AND assisted | 37 |
| #34. | reproductive AND technic, AND assisted | 8 |
| #33. | assisted AND reproductive AND technic | 8 |
| #32. | assisted AND reproductive AND technics | 37 |
| #31. | techniques, AND assisted AND reproductive | 6,811 |
| #30. | technique, AND assisted AND reproductive | 3,505 |
| #29. | reproductive AND technique, AND assisted | 3,505 |
| #28. | assisted AND reproductive AND technique | 3,505 |
| #27. | reproductive AND techniques, AND assisted | 6,811 |
| #26. | #12 OR #13 OR #14 OR #15 OR #16 OR #17 OR #18 OR #19 OR #20 OR #21 OR #22 OR #23 OR #24 OR #25 | 77,063 |
| #25. | 'test tube' AND baby | 228 |
| #24. | test AND tube AND babies | 245 |
| #23. | baby, AND 'test tube' | 228 |
| #22. | babies, AND 'test tube' | 107 |
| #21. | 'test tube' AND babies | 107 |
| #20. | fertilizations AND in AND vitro | 144 |
| #19. | 'test tube' AND fertilizations |  |
| #18. | test AND tube AND fertilization | 658 |
| #17. | fertilizations, AND 'test tube' |  |
| #16. | fertilization, AND 'test tube' | 294 |
| #15. | 'test tube' AND fertilization | 294 |
| #14. | in AND vitro AND fertilizations | 144 |
| #13. | in AND vitro AND fertilization | 76,705 |
| #12. | fertilization AND in AND vitro | 76,705 |
| #11. | #1 OR #2 OR #3 OR #4 OR #5 OR #6 OR #7 OR #8 OR #9 OR #10 | 91,898 |
| #10. | luteoziman | 1 |
| #9. | luteozyman | 1 |
| #8. | interstitial AND cell AND stimulating AND hormone | 475 |
| #7. | hormone, AND interstitial AND 'cell stimulating' | 119 |
| #6. | interstitial AND 'cell stimulating' AND hormone | 119 |
| #5. | icsh AND interstitial AND cell AND stimulating AND hormone | 33 |
| #4. | lh AND luteinizing AND hormone | 46,499 |
| #3. | lutropin | 1,013 |
| #2. | hormone, AND luteinizing | 91,416 |
| #1. | luteinizing AND ('hormone'/exp OR hormone) | 91,416 |


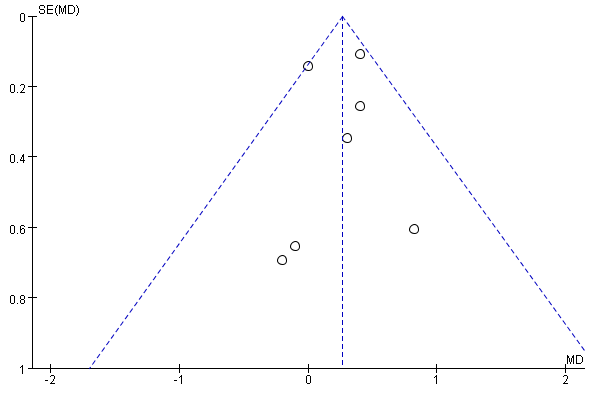


**Supplementary Fig. 1 Funnel plot of EMT on trigger day.** SE, standard error. MD, mean difference. No asymmetry was found in the funnel plot.


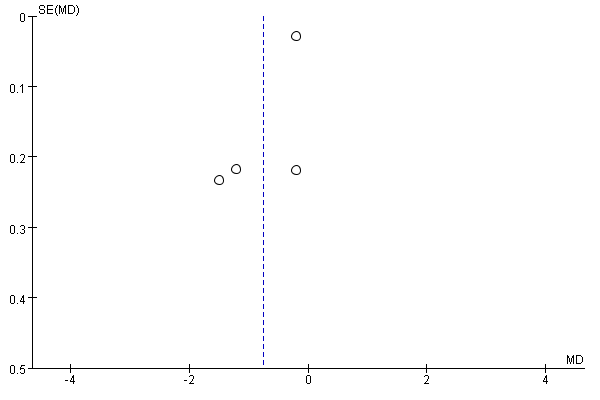


**Supplementary Fig. 2 Funnel plot of number of high-quality embryos**. SE, standard error. MD, mean difference. No asymmetry was found in the funnel plot.

**Supplementary Fig. 3 Egger’s publication bias plot of EMT on trigger day.**

**Supplementary Fig. 4 Egger’s publication bias plot of number of high-quality embryos**


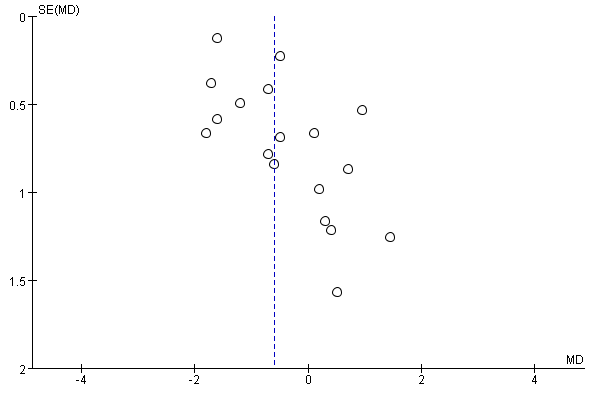


**Supplementary Fig. 5 Funnel plot of number of oocytes retrieved.** SE, standard error. MD, mean difference. Asymmetry was found in the funnel plot.

**Supplementary Fig. 6 Egger’s publication bias plot of number of oocytes retrieved.**
